# Differential expression of conditioned medium from tauopathies patients' olfactory neuroepithelial precursor cells, implications in the neurodegeneration

**DOI:** 10.1002/alz70855_104103

**Published:** 2025-12-24

**Authors:** Obed‐Ricardo Lora‐Marin, Juan‐Ramon Padilla‐Mendoza, Daniela‐Elizabeth Gomez‐Ramirez, Maria‐del‐Carmen Cardenas‐Aguayo

**Affiliations:** ^1^ Neurosciences Master in Science Program INB, UNAM, CDMX, DF, Mexico; ^2^ UNAM, School of Medicine, Department of Physiology, CDMX, DF, Mexico; ^3^ División de Químico Biológicas, Universidad Tecnológica de Tecámac, Mexico, EM, Mexico

## Abstract

**Background:**

In neurodegenerative diseases such as tauopathies, including Alzheimer's disease dementia (AD) and progressive supranuclear palsy (PSP), cellular processes such as survival, proliferation, and cellular communication are highly affected. These alterations are linked to changes in the expression of various trophic factors. Various cellular models are employed to study certain tauopathies, including peripheral cells. An innovative model involves human olfactory neuroepithelial progenitor cells (hONE‐NPCs). Therefore, analyzing and characterizing the trophic factors released by hONE‐NPC‐AD and hONE‐NPC‐PSP could provide insights into the alterations occurring during the progression of these diseases (Lampinen et al., 2022).

**Method:**

Through immunodetection, cells from Mexican healthy controls from two age groups (Age 30‐40 and 50‐60 years old) and Mexican patients with Alzheimer's disease dementia and progressive supranuclear palsy were characterized. Conditioned media were first purified and concentrated using centrifugation and acetone precipitation. Subsequently, electrophoresis and Coomassie blue staining were performed to identify trophic factors present in the conditioned media through mass spectrometry.

**Result:**

hONE‐NPCs expressed proteins such as Ki67, Nestin, GFAP, and b‐III Tubulin, which are associated with cellular proliferation and the neuronal progenitor lineage. The functional characterization of hONE‐NPCs in AD and PSP patients revealed duplication times of approximately 90 hours. In the conditioned media, a differential expression of trophic factors released by hONE‐NPCs from AD and PSP patients was observed compared to apparently healthy age‐matched controls and young controls.

**Conclusion:**

The differential expression of trophic factors released by hONE‐NPCs in AD and PSP patients is significant compared to apparently healthy participants. These trophic factors are linked to signaling pathways involved in cellular proliferation and survival. This reduction directly impacts cellular proliferation and survival, as demonstrated by phenotypic characterization, showing decreased Ki67 expression relative to the total number of DAPI‐stained cells. To date, no studies have focused on the conditioned media or the changes in trophic factors during tauopathies, such as Alzheimer's disease dementia and PSP, in primary cultures of human olfactory neuroepithelial progenitor cells, opening a new research avenue.

